# Biosensors for Detection of Labile Heme in Biological Samples

**DOI:** 10.3390/bios16010004

**Published:** 2025-12-19

**Authors:** Krysta Dobill, Delphine Lechardeur, Jasmina Vidic

**Affiliations:** INRAE, AgroParisTech, Micalis Institute, Université Paris-Saclay, 78350 Jouy-en-Josas Cedex, France

**Keywords:** heme, hemoglobin, heme biosensors, hemolysis, heme synthesis, heme regulation, whole-cell biosensor, heme detection

## Abstract

Heme, a protoporphyrin IX iron complex, functions as an essential prosthetic group in hemoglobin and myoglobin, mediating oxygen storage and transport. Additionally, heme serves as a critical cofactor in various enzymes such as cytochrome c, enabling electron transfer within the mitochondrial respiratory chain. Unlike protein-bound heme, free or labile heme exhibits cytotoxic, pro-oxidant, and pro-inflammatory properties. Elevated levels of free heme are associated with various pathophysiological conditions, including hemolytic disorders such as sickle cell disease, malaria, and sepsis. In this review, we introduce the physiological roles of heme and its involvement in human health and disease. We also examine the mechanisms of heme sensing and regulation in bacterial cells. A variety of analytical methods have been developed to detect and quantify heme, enabling differentiation between protein-bound and free forms. These tools are discussed in the context of their applications in studying cellular heme regulation and their use in monitoring pathological conditions in humans. In particular, we describe examples of biosensors employing bacterial heme sensor proteins as recognition elements.

## 1. Introduction

Heme belongs to the large family of modified tetrapyrrole molecules with diverse conjugation and metal chelation systems. It is a protoporphyrin coordinated to an iron atom and is the most abundant form of iron in vertebrates. It gives blood its red color, where it is associated with hemoglobin (Hb) in erythrocytes [1,2]. Moreover, heme serves as a prosthetic group in several enzymes, hemoproteins, that are involved in many functions [3]. The ability of iron to exist in multiple oxidation states enables redox reactions in enzymes such as peroxidases, catalases, and cytochromes, as well as the transport of diatomic gases, as seen in hemoglobins and myoglobins. Independent of its catalytic role, by binding to sensor proteins, heme can also function as a signalling molecule, regulating various processes such as cellular responses to O_2_ or CO and the activity of transcription factors [1,4,5,6].

Protein-free, also called “free” or “labile”, heme is extremely toxic because of its reduced form of iron (Fe^2+^). The ferrous ion is highly reactive, particularly with oxygen [7,8] and can participate in the production of free radicals via the Fenton reaction:

Fe^2+^ + H_2_O_2_ → Fe^3+^ + OH^−^ + HO

The hydroxyl radical is a very powerful oxidant, leading to oxidative stress that can degrade DNA, lipids and proteins, damage cells, and cause hemolysis and inflammation [9,10]. Furthermore, as heme is lipophilic, it accumulates in cell membranes, where it directly damages membrane lipids and proteins [9,10]. Furthermore, as heme is lipophilic, it accumulates in cell membranes, where it directly damages membrane lipids and proteins [10,11,12,13]. The labile pool of protein-free heme present in circulating plasma is a potential oxidative threat. This can occur upon degradation of hemoglobin, myoglobin, or heme-containing enzymes. To safeguard the organism, plasma proteins, including haptoglobin, hemopexin, serum albumin, and high- and low-density lipoproteins, sequester labile heme to neutralize its toxicity and ensure its clearance by the liver and macrophages [5,14,15]. However, in some pathologies, clearance is poor due to abnormal lysis of erythrocytes, resulting in the presence of extracellular free heme in biological samples such as blood or plasma, feces, or urine. These pathologies require the quantitative detection of heme, which remains challenging because detection must be performed in highly complex biological matrices, and labile heme must be distinguished from that bound to proteins.

In this review, we briefly summarize the synthesis and biological functions of heme and its involvement in certain human pathologies. We then focus on bacterial sensors for heme detection. Finally, we highlight recent advances in analytical methods for heme detection and quantification, particularly focusing on biosensor-based techniques.

## 2. Biological Functions of Heme

**Figure 1:**
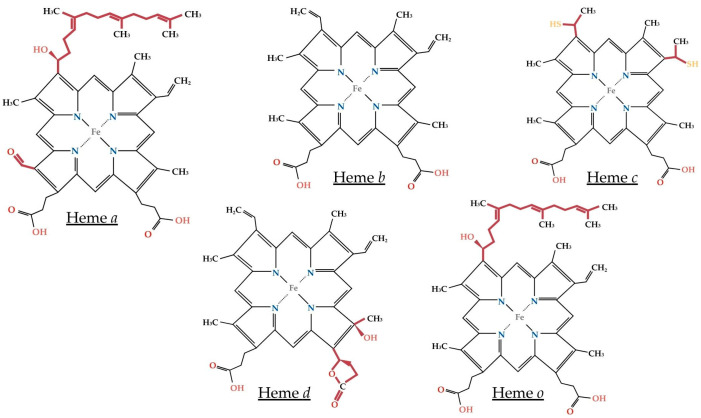
Chemical structures of naturally occurring heme molecules *a*, *b*, *c*, *d* and *o*. Heme *b* is the most abundant form, but some ramifications around the porphyrin ring (in red) generate other forms of heme used by different types of organisms. These forms are implicated in diverse enzymatic reactions.

Heme is an iron atom coordinated to a porphyrin core composed of four pyrrole rings (a tetrapyrrole) linked by methene bridges (Figure 1). Heme *b* is the most widespread in the human body, as it is a component of hemoglobin, myoglobin, and some cytochromes and peroxidases. Heme *b* plays an important role in oxygen transport, signaling, electron transfer, transcription, and catalysis [16]. The enzyme heme o synthase converts heme *b* into heme *o*, which can be further converted to heme *a* by the a synthase [17]. Heme *a* contains a formyl group on ring D instead of a methyl group as in heme *b* (Figure 1). As prosthetic groups, hemes *b*, *a*, and *o* are tightly, but non-covalently, bound within their host proteins. Heme *c*, also known as cytochrome *c*, is formed from heme *b* through the replacement of vinyl groups by thioether groups and covalent attachment to the protein backbone [18]. Heme *d* is much less abundant in nature and is found in the cytochrome *cd1* enzyme, responsible for the reduction of nitrite to nitric oxide in some bacteria [19], or acts as a cofactor in the terminal oxidase that predominates in *Escherichia coli* at low oxygen levels [20]. The generic term heme will refer to heme b in its reduced or oxidized form for the remainder of the article.

### 2.1. Heme Interaction with Proteins

**Figure 2:**
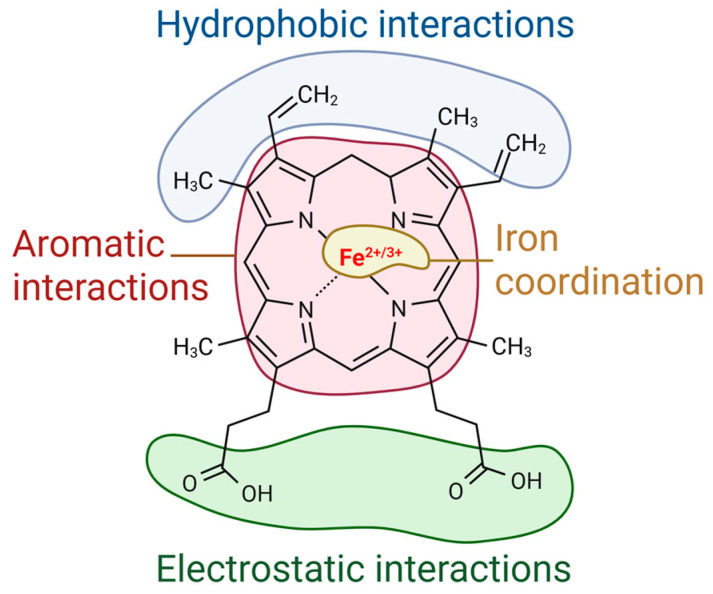
Schematic representation of the broad diversity of interactions that heme can form with proteins. Illustration adapted from [22] and created in BioRender. Vidic, J. (2025) https://BioRender.com/584oc2v.

Heme can interact with various molecules through a range of covalent and non-covalent binding modes [21,22]. The cyclic porphyrin ring of heme enables aromatic interactions with cyclic aromatic amino acids such as phenylalanine, tyrosine, and tryptophan, while the hydrophobic vinyl and methyl groups in the porphyrin core favor hydrophobic van der Waals interactions with hydrophobic amino acids such as leucine, isoleucine, and valine [23]. Figure 2 shows regions of the heme that can be involved in different types of interactions. Ionic and hydrogen bonds can also form between the propionate groups of heme and positively charged amino acids such as arginine, histidine, or lysine [23].

Heme iron is coordinated with four nitrogen atoms of the porphyrin ring, as shown in Figure 1. Additionally, this iron can bind to one or two other ligands, becoming pentacoordinated or hexacoordinated, respectively. When heme is pentacoordinated, the sixth bond is usually formed with a non-protein molecule such as a solvent (like water) or a diatomic gas (such as O_2_, CO, or NO) [24,25,26] (Figure 2). Some atoms in the side chains of amino acids, such as sulfur, nitrogen, or oxygen, can act as iron ligands. This is the case for histidine, which is the coordinating amino acid in most hemoproteins, as well as cysteine, tyrosine, and methionine [22]. The amino-acid side-chains coordinated to the iron atom do not directly interact with substates in enzymatic reactions. The protein domain responsible for heme binding often contains a hydrophobic pocket around the amino acid coordinating the iron, which interacts with the porphyrin core. Finally, certain types of heme can bind covalently to proteins via the side chains of the porphyrin core [27]. This is the case for heme *c*, which can form thioester bonds with cysteines

### 2.2. Heme-Mediated Oxygen Transport: Hemoglobin

The vast majority of heme in vertebrates is heme *b*, which is non-covalently associated with hemoglobin (Hb) and myoglobin in blood and muscle cells, respectively. The function of Hb is to transport oxygen in the erythrocytes of nearly all vertebrates, as well as in the tissues of some invertebrates [28,29,30]. Packaging Hb at high density in erythrocytes (30–35 g/mL; 97% of the erythrocyte dry content) promotes the stable tetrameric assembly of Hb and helps maintain heme iron in its reduced state. It also prevents the high concentration of this protein from affecting blood viscosity and osmolarity [31]. The erythrocyte membrane prevents the reactive heme molecule from contacting the environment.

**Figure 3:**
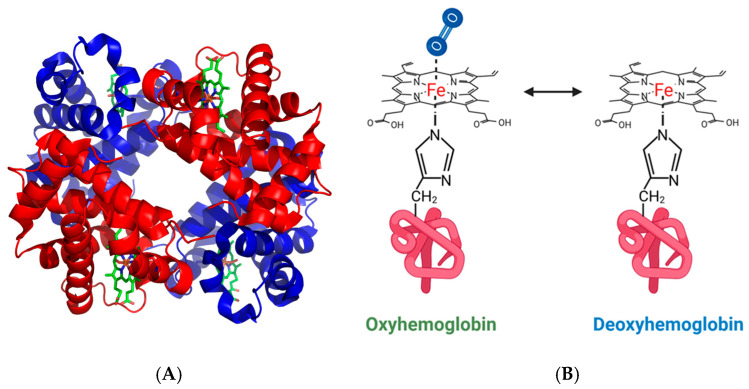
Heme interaction with hemoglobin. (**A**) Crystal structure of the tetrameric hemoglobin formed by 2 α (in red) and 2 β subunits (in blue). Each monomer binds to one heme molecule (in green) (from PDB: 1GZX Proteopedia Hemoglobin) [2]. (**B**) Schematic representation of oxygen binding to hemoglobin. Heme iron coordinates both to a hemoglobin histidine and to O_2_ as the sixth ligand.

Human Hb consists of two α and two β subunits, with a similar three-dimensional structure; each subunit binds one molecule of heme (Figure 3A). Upon oxygen binding, Hb undergoes substantial quaternary structural rearrangements that underlie the cooperative binding of its four oxygen molecules. The capacity of Hb-heme to bind diatomic gases is dictated by the oxidation-reduction state of the heme iron. In erythrocytes, heme iron is maintained in the reduced state (Fe^2+^) by intracellular reductases [31]. In these conditions, the fifth coordination site is occupied by the imidazole ring of a histidine, and the sixth position by O_2_, CO, NO, or isocyanides [32]. In deoxyhemoglobin, the sixth coordination site remains unoccupied (Figure 3B). Hemoglobin carries O_2_ from the pulmonary capillaries to the organs and also ensures about 10% of CO_2_ elimination.

In humans, erythrocytes function in the blood for three months before being degraded by macrophages in the bloodstream (approximately 200 billion erythrocytes reach senescence daily). Damaged and senescent erythrocytes are detected by specialized erythrophagocytic macrophages in the spleen, liver, and bone marrow, which remove these cells before they undergo hemolysis. In erythrocytes, the high affinity of heme for apohemoglobin is optimized by the reducing cytoplasm, which maintains the iron in heme in the ferrous form. In the plasma, Hb and heme are recognized by specific circulating proteins that neutralize the toxicity of labile heme [15,21].

## 3. Heme-Related Blood Disorders

Labile heme as a cofactor is ubiquitous in cells, which must ensure its timely bioavailability while minimizing cytotoxicity caused by its redox activity. The inability to control the concentration of labile heme occurs in some life-threatening diseases [33]. Heme toxicity is well documented because of its impact on human health, especially its pro-inflammatory and pro-thrombotic effects [34], as well as its disruption of the immune system, which promotes bacterial co-infection [35]. Heme iron generates toxic reactive oxygen species (ROS) and binds to NO, thus interfering with its function as a vasodilator [36,37]. Free heme also activates the DAMP (damage-associated molecular pattern) receptor TLR4 and initiates the NFκB signalling pathway, leading to the synthesis of pro-inflammatory cytokines such as TNF-α and IL-10 [38]. Free heme thus triggers inflammation and damages the endothelial cells of blood vessels.

Various causes can lead to hemolytic diseases, such as alloimmune hemolysis, drug- and toxin-induced hemolysis, and autoimmune hemolytic anemia. The synthesis of an abnormal hemoglobin protein (hemoglobin S or HbS) due to a mutation in the β-globin gene is a genetic and hereditary disorder called sickle cell disease (SCD). SCD is characterized by a crescent-shaped morphology of erythrocytes. Moreover, deoxygenated HbS is prone to polymerization, leading to increased rigidity of erythrocytes and making them more susceptible to hemolysis [39]. SCD affects about three million people, mainly of African, Mediterranean, Middle Eastern, and South Asian descent [40]. Another pathology, thalassaemia, encompasses several anemic diseases caused by defects in hemoglobin synthesis. Mutations in the genes coding for hemoglobin chains (α and β) cause an imbalance between the α and β chain ratio [41]. As a result, abnormal Hb is prone to aggregation, leading to two major consequences: abnormal proliferation and differentiation of red blood cells (ineffective erythropoiesis). Excess heme and aggregated Hb are synthesized but are not efficiently incorporated into erythrocytes, leading to heme-catalyzed ROS production [41]. Moreover, defective erythrocytes are more likely to lyse in the patient’s bloodstream [42]. Porphyria refers to a group of eight disorders caused by mutations in one of the eight enzymes involved in the heme biosynthesis pathway. All porphyrias are characterized by extremely high levels of heme intermediates in the plasma, bile, and urine [43]. Porphyrias are hereditary except for porphyria cutanea tarda, which in more than 80% of cases is acquired through lifestyle factors such as smoking, alcohol abuse, and viral infections (for example, hepatitis C or HIV) [44].

A high level of labile heme is also associated with sepsis or malaria. Sepsis refers to the dysregulation of the immune system following an infection, causing multiple organ failure [45]. Over-induction of several immune signalling pathways leads to the production of pro-inflammatory cytokines, which induce uncontrolled coagulation [46]. Erythrocytes are torn apart while trying to circulate in the coagulated blood vessels, leading to mechanical hemolysis. Bacteria and fungi associated with sepsis can also produce haemolysins as virulence factors [47,48]. Malaria is an infectious disease caused by the parasite *Plasmodium falciparum*. With almost 250 million cases a year, malaria is the most prevalent parasitic disease affecting humans, according to the World Health Organization [49]. During the infection cycle, the parasites use the bloodstream to reach the liver, where they begin their first maturation step in the hepatocytes [35]. After this stage of development, the parasites invade erythrocytes to mature into adult forms. Mature parasites induce hemolysis of the infected erythrocytes to start a new reproductive cycle [35]. Haemolysis causes anaemia, which is the leading cause of death in infected patients [49].

Colon cancer is one of the most prevalent cancers in both men and women worldwide. Early detection is performed by screening faeces for the presence of Hb or its breakdown product heme, because colon cancer is frequently characterised by abnormal bleeding in the gastrointestinal tract [50,51].

## 4. Heme Biosensing in Bacteria

In bacteria, the catalytic functions of heme are essentially the same as in eukaryotic cells: heme is a cofactor for enzymes such as cytochrome oxidases, peroxidases, and catalases. Control of heme homeostasis is crucial for bacterial survival to limit its toxicity, especially in Gram-positive bacteria [52,53,54,55,56,57,58]. Over the course of evolution, bacteria have developed different strategies to control heme toxicity, with the two main ones being efflux and sequestration. Heme degradation by heme oxygenase also contributes to reducing heme toxicity by cleaving the porphyrin core and allowing iron recycling [59,60,61,62].

Recently, heme was also shown to function in signaling pathways that activate mechanisms protecting bacteria from the toxicity of extracellular heme. Heme binds to transcription factors or two-component systems, inducing conformational changes and consequently modulating their activity [63,64,65,66]. These heme biosensor proteins control the transcription of proteins involved in heme homeostasis, allowing bacterial adaptation according to heme concentration.

### 4.1. HsmR

In *Clostridium difficile*, HsmRA (Hsm, heme-sensing membrane protein; R, regulator; A, membrane protein) consists of a transcription inhibitor from the MarR family (HsmR) [67]. Upon heme binding, HsmR is released from its target promoter sequence, activating the transcription of a membrane protein (HsmA) capable of sequestering heme [67]. This interaction has two identified functions: first, to reduce heme toxicity by lowering its concentration, and second, to increase resistance to compounds that generate oxidative stress, such as vancomycin or metronidazole. The mechanism of detoxification of oxidative stress and antibiotics remains to be elucidated.

### 4.2. HatR and the Hat Efflux System in Clostridioides Difficile

Efflux of excess heme is a common strategy in many bacteria to limit its toxicity. Several mechanisms have been described for Gram-positive bacteria.

The Hat (heme-activated transporter) heme efflux system, which is strongly induced by heme, was identified during transcriptomic analysis of *Clostridioides difficile* grown in heme-supplemented conditions [68]. The system comprises a two-gene *hatRT* operon, including a transcriptional regulator, HatR from the TetR family, and a transporter from the Major Facilitator Superfamily (MFS), HatT [68]. These membrane permeases are known to transport a variety of compounds such as sugars, amino acids, nucleosides, and antibiotics. HatR functions as transcriptional repressor of the *hatRT* operon when heme is absent [68]. When heme is present, HatR binds to heme, and the resulting HatR-heme complex detaches from the DNA, allowing transcription of the operon.

### 4.3. PefR and Pef Efflux Transport

The Pef (porphyrin efflux) transporter system was first described in *Streptococcus agalactiae* through a transcriptomic study, which showed that two operons, *pefAB* and *pefRCD*, were strongly induced in the presence of heme [63]. This system includes a transcriptional regulator PefR from the MarR (multiple antibiotic resistance regulator) family of repressors, which controls both operons, and two transporters from the MFS (Major Facilitator Superfamily), PefAB and PefCD. MarR family regulators are involved in stress response, virulence, or the export of toxic products such as antibiotics. Without heme, PefR binds to the promoters of the two operons, *pefAB* and *pefRCD*, preventing their transcription. Interestingly, the corresponding PefCD transporter from the group A Streptococcus *Streptococcus pyogenes* is specific for protoporphyrin IX but not for heme or its derivatives [63]. The PefCD transporters in *Sterptococcus suis* and *Lactococcus lactis* also happen to be located near their respective multidrug transporters SatAB and LmrCD [69].

### 4.4. Heme Biosensors Regulating HrtBA Heme Efflux Transporter

The Hrt (heme-regulated transporter) system was first described in *Staphylococcus aureus*. A proteomic study of the impact of heme in *S. aureus* showed that the *hrtBA* operon was strongly induced in the presence of heme [70,71]. This operon is composed of two genes: *hrtA*, which encodes an ATPase, and *hrtB*, which encodes a transmembrane permease, together forming an ABC transporter. Homologous proteins are also found in a series of Gram-positive bacteria (Lactococci, Streptococci, Enterococci, as examples) [71]. Interestingly, HrtBA transcription is controlled by distinct heme sensor proteins, depending on the bacterial species.

#### 4.4.1. HrtR (Heme-Regulated Transport Regulator) in *Lactococcus lactis*

The dimeric HrtR is a cytoplasmic transcriptional regulator belonging to the TetR family, involved in regulating *hrtBA* transcription in *Lactococcus lactis*. The *hrtR* gene is integrated into the *hrtRBA* operon, which is strongly induced by heme [64]. Its deletion results in hypersensitivity of the strain to heme toxicity [64]. In the absence of heme, HrtR represses transcription of the *hrtRBA* operon by binding to a 15-nt palindromic sequence via an arginine (R46) and a tyrosine (Y50) [72]. One heme iron binds to each monomer of HrtR via coordination to H72 and H149 in a hydrophobic pocket [64]. The crystallographic structure of the HrtR-heme complex revealed a conformational change caused by heme binding, which induces the release of the HrtR-heme complex from DNA [72].

#### 4.4.2. FhtR (Faecalis heme Transport Regulator) in *Enterococcus faecalis*

FhtR, a transcriptional inhibitor of the TetR family, functions similarly to HrtR. FhtR is a protein that binds heme with a pentacoordinate coordination of the iron, involving Y132, which appears to play a crucial role in heme sensing [65]. FhtR directly regulates *hrtBA* expression by binding to two 14-nt palindromic sequences in the *hrtBA* promoter, repressing its transcription. When heme binds to FhtR, the heme/FhtR complex loses its affinity for the promoter, allowing transcription of the *hrtBA* operon.

#### 4.4.3. HssS in *S. aureus*

**Figure 4:**
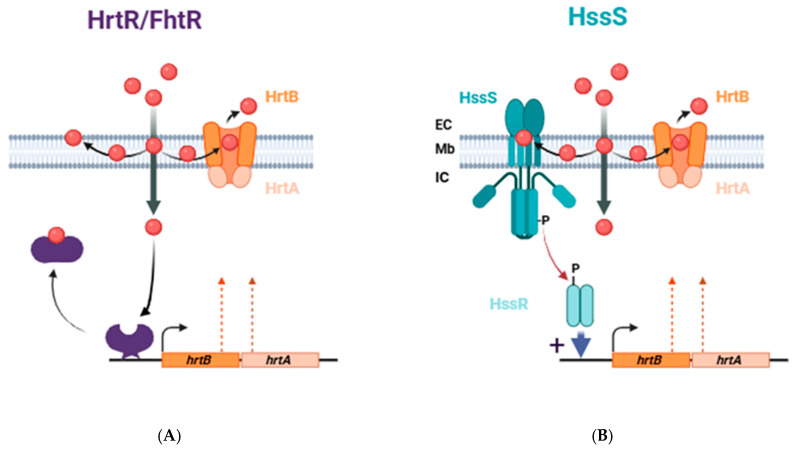
Heme-sensing mechanisms regulating the expression of the HrtBA heme efflux transport system. (**A**) In commensal and food Gram-positive bacteria, *hrtBA* transcription is controlled by heme biosensor-protein acting as transcriptional inhibitors (HrtR in *L. lactis*; FhtR in *E. faecalis*). Upon binding to heme, these biosensors lose their affinity to a specific DNA sequence in the *hrtBA* promoter. (**B**) In *S. aureus* and other Gram-positive pathogens, heme from the environment is detected by the histidine kinase sensor HssS. The heme-binding domain is located at the interface between the extracellular environments. Heme binding induces the phosphotransfer from the sensor to the response regulator HssR, which activates the transcription of *hrtBA*. Adapted from [64,65,66].

In *S. aureus*, the system involved in hrtBA regulation is called HssRS (heme sensor system) and encodes the two proteins of a two-component system (2-CS): HssS, a histidine kinase (HK), and HssR, a response regulator, as shown in Figure 4 [66]. HssS is a 457-amino acid membrane protein with two transmembrane domains connected by a short extracellular loop (135 amino acids) and a cytoplasmic region composed of conserved domains: a Histidine Kinase, Adenylate Cyclase, Methyl-accepting protein, and Phosphatase (HAMP) domain, a Dimerization Histidine phosphotransfer (DHp) domain, and a catalytic (CA) site. H249 of the DHp domain is the amino acid autophosphorylated by the autokinase activity of HssS. Phosphorylation is then transferred to D 52 of HssR, enabling activation of the regulator. A recent study shows that heme binds to *S. aureus* HssS in a hydrophobic pocket at the interface between the membrane and the cytoplasmic domains [66]. Phosphorylated HssR binds to a repeated DNA sequence (2 × 9 nucleotides) upstream of the TATA box and the RNA polymerase binding site. HssR binding activates transcription of the *hrtBA* genes and production of the transporter. The two amino acids (H249 of HssS and D52 of HssR) are essential for 2-CS function, and mutation of either prevents signal transduction.

## 5. Diagnostic Methods

Although only hemoglobin is commonly included in health check-ups as an indicator of general health, accurately measuring the labile heme pool in cells is vital for understanding and managing certain diseases. Measuring labile heme is important because this chemically reactive, biologically active pool of unbound heme acts as a potent pro-oxidant that drives oxidative stress and cellular damage, serves as a signaling molecule regulating gene expression and inflammatory pathways, and reflects the state of heme homeostasis within cells. Its levels change significantly in diseases involving hemolysis and inflammation, such as malaria, sickle cell disease, sepsis, and other conditions, where excess labile heme contributes to pathology. Monitoring labile heme also helps clarify host–pathogen interactions, particularly in heme-rich environments like *Plasmodium falciparum* infection, and supports the development and evaluation of therapeutic strategies aimed at limiting heme toxicity or modulating heme-dependent biological processes. To address those needs, various methodologies and biosensors have been developed.

### 5.1. Instrument-Based Methods for Heme Detection

The most frequently used heme detection methods measure the absorption intensity of the hemochromogen complex using a spectrophotometer. The hemochromogen forms when heme in its reduced Fe^2+^ state binds to pyridine, which induces a significant increase in absorption intensity at 557 nm [73]. Such direct tests have serious drawbacks due to their lack of specificity and uncertain quantitative interpretation. This test does not distinguish between free heme and covalently bound heme. Moreover, it can also be used to measure the amount of heme associated with a protein, since pyridine has a very high affinity for heme via affinity transfer. The sensitivity of spectrophotometric heme determination was improved by using the pseudo-peroxidase activity of heme and the Turbo TMB (3,3′,5,5′-tetramethyl-benzidine) substrate used in ELISA tests [74]. This method was validated by monitoring the absorbance of oxidized TMB at 385 nm, which increased proportionally to the concentration of free heme. In another study, Atamna et al. [75] measured the reconstitution of apo-horseradish peroxidase (apo-HRP) with heme to form holo-HRP. The resulting holo-HRP activity was measured at 652 nm upon addition of a colorimetric substrate. The reaction was specific, as they showed that apo-HRP specifically binds free heme but not heme bound to housekeeping heme-proteins. More recently, cysteine was used as a spectroscopic probe since cysteine shows a distinct spectral effect upon interacting with free heme [76]. The concentration of free heme was measured by the absorbance maximum at 364 nm, which is typical for the interaction of cysteine with free hemin. Heme is the biologically active ferrous form (Fe^2+^), while hemin is its more stable ferric form (Fe^3+^) commonly used in laboratory experiments. The method showed low sensitivity, with a detection limit of 0.5 µM. However, this technique, when applied in serum, allowed estimation of the heme-binding capacity of scavenging proteins such as albumin, hemopexin, and immunoglobulins.

To determine heme levels in bacteria and fungi grown under different culturing conditions that promote microbial synthesis and/or acquisition of heme, Fyrestam et al. [77] coupled high-performance liquid chromatography (HPLC) with tandem mass spectrometry (MS/MS). The detection limit for the method, determined by injection of pure hemin, was 0.75 pmole. When this HPLC-MS/MS method, combined with liquid–liquid extraction, was applied to Saccharomyces cerevisiae and Escherichia coli as model organisms, it was shown that the addition of FeSO_4_, hemin, 5-aminolevulinic acid hydrochloride, and protoporphyrin IX cobalt chloride affected heme and iron acquisition mechanisms. Moreover, they demonstrated that cobalt protoporphyrin IX could mimic heme, and its uptake reduced intracellular heme concentrations in E. coli. These findings suggest that microbial heme acquisition pathways may serve as potential targets for the treatment of bacterial infections.

Traditional methods, such as HPLC and HPLC-MS, for detecting the composition and dynamics of labile heme pools are not widely available because they rely on bulky instrumentation. Considering the high importance of labile heme under both physiological and disease states, biosensors have been increasingly developed as alternative analytical tools that can quantify the heme pool. Table 1 presents examples of biosensors reported in the literature that can distinguish between protein-bound and labile heme.

### 5.2. Optical Biosensors for Heme Detection

**Figure 5:**
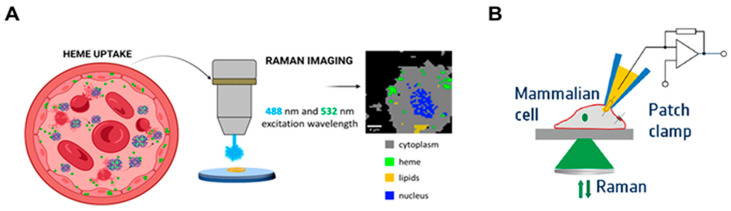
Raman spectroscopy for heme detection and subcellular localization. (**A**) The experimental scheme of visualization of the heme uptake by endothelial cells using Raman imaging at two wavelengths to achieve heme sub-cellular localization. Adapted with permission from [80]. (**B**) Combination of patch clamp and Raman spectroscopy. A HEK 293 cell contacted by a patch-clamp pipette filled with hemin. After internalization, hemin can diffuse into the whole cell. The Raman Spectroscopy provides access to the cell interior and quantifies free and protein-bound hemin. Adapted with permission from [82].

Raman spectroscopy and imaging have demonstrated exceptional performance as analytical tools for quantifying heme and heme proteins in biological systems [78,79]. Raman and Resonance Raman spectroscopy offer high sensitivity, enabling the detection of minimal structural modifications in free or protein-bound heme groups during cellular processes. Moreover, Resonance Raman spectroscopy allows tracking of the heme active site environment, differentiation between Fe^2+^ and Fe^3+^, and detection of porphyrin ring plane movements in response to changes in oxygenation states [78]. Label-free Raman imaging using two different excitation wavelengths enabled detection of heme uptake by endothelial cells and characterization of the oxidation state of the iron ion in intercalated heme in situ (Figure 5A). This approach was successfully applied to visualize heme uptake in mice with developed atherosclerosis [80]. Similarly, Raman imaging using two excitation wavelengths (488 and 532 nm) allowed tracking of heme and its iron recycling process inside murine Kupffer and macrophage cells [81].

Neugebauer et al. [82] combined patch clamp access to the cytosol of HEK 293 cells, loading them with hemin (>5 µM), with Raman spectroscopy to detect molecular changes due to hemin binding to cellular components (Figure 5B). Distinct Raman bands were obtained for free and protein-bound hemin, as well as for low-spin and high-spin hemin, without any labeling [82]. The same group applied non-invasive, label-free, and nondestructive resonance Raman spectroscopy using excitation wavelengths ranging from 413 nm to 752 nm to estimate the detection limits for hemin, myoglobin, biliverdin, and bilirubin [83]. The detection limits were in the range of 20–100 μM.

Surface-Enhanced Raman Scattering (SERS) is an enhanced form of Raman spectroscopy that provides signal intensities many orders of magnitude higher by using special nanostructured metal surfaces, such as silver colloids [84]. It is a powerful analytical technique used to detect and analyze molecules at very low concentrations, often down to single-molecule sensitivity [84]. However, SERS measurement of intracellular heme can be compromised by the presence of interfering molecules such as cytochrome C, glutathione, and DNA. To address these nonspecific interactions, SERS was combined with droplet-based microfluidics. The microfluidic lab-on-a-chip device enabled detection of hemin in pure solution or mixtures through a segmented flow of aqueous droplets and showed potential to differentiate between protein-bound and free heme [83]. To achieve this, SERS measurement positions were fixed while the segmented flow provided continuous movement of the droplets during detection.

Raman-based biosensors, especially SERS platforms, offer ultra-high sensitivity and molecular specificity, but they also have several important drawbacks for clinical implementation. Raman requires strong enhancement or high-power lasers, which may photodamage biological samples. Biological samples have significant fluorescence backgrounds, which reduce signal clarity for in vivo sensing. Additionally, water absorption and tissue scattering can restrict the effective depth of Raman detection, limiting in vivo applications.

Spectroscopic approaches are popular because of their assay simplicity [85,86]. For example, to detect free heme at low concentrations, an optical biosensor employing a fluorescently labeled heme oxygenase-1 enzyme was used [87]. Heme oxygenase-1 catalyzes the oxidative degradation of heme and binds it with high affinity. The assay was based on fluorescence quenching that occurs upon heme binding to the enzyme. In this way, the reduction in fluorescence upon heme binding to the labeled protein provided a quantitative measure of heme concentration. Although easy to perform, spectrophotometric methods cannot be applied directly to complex samples such as urine, feces, and blood plasma, but require time-consuming sample preparation to separate heme from the biological matrices. Moreover, fluorescent or colorimetric biosensors rely on external labeling and have relatively large measurement errors. Therefore, they cannot compete with commercial hematology analyzers and are not employed in clinical heme measurement.

Optical techniques, particularly spectrophotometry, are the established reference methods for quantifying heme in controlled laboratory settings. However, the development of optical biosensors for direct, point-of-care heme detection in complex biological matrices is significantly constrained by matrix-induced optical interference. The primary challenges include the profound inner-filter effect from hemoglobin itself at high concentrations, spectral overlap from competing chromophores (e.g., bilirubin), and intense light scattering from particulates in stool or cells in blood, which collectively compromise sensitivity and accuracy. This underscores a critical design imperative: future optical biosensor platforms aiming for direct heme detection in clinical samples must either incorporate effective on-chip sample clarification and analyte purification or exploit advanced optical techniques.

**Figure 6:**
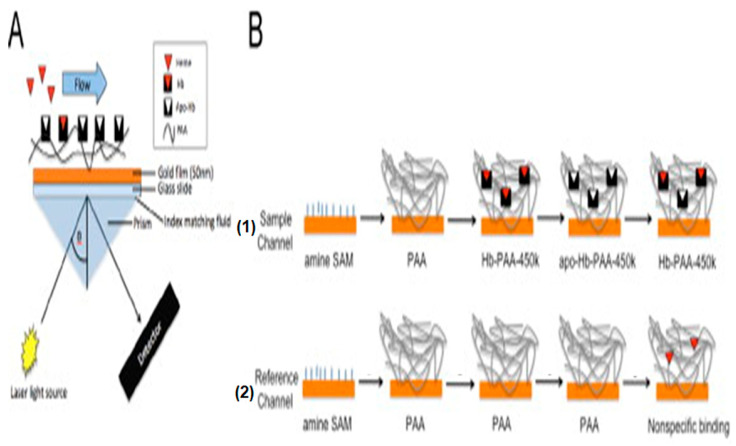
Principle of the SPR biosensor for heme detection. (**A**) Schematic illustration of the SPR biosensor. (**B**) Sample channel was obtained by immobilization of apohemoglobin via an amino link to a polyacrylic acid (PAA) layer over gold surface. The control channel was modified only PAA. Injection of heme solution over each channel was quantified after subtraction of nonspecific binding estimated on the control channel. Adapted with permission from [88].

In addition, colorimetric and fluorescent spectroscopic biosensors cannot provide real-time or automated analysis. To overcome these limitations of spectroscopic methods, Briand et al. [88] proposed a surface plasmon resonance (SPR) biosensor for heme detection. Plasmonic biosensors, based on an optical phenomenon created by light interaction with conducting interfaces in thin films or nanoparticles that are smaller than the incident wavelength, are among the most used devices due to their relatively low cost and ease of use [84]. The SPR signal was generated upon selective binding of heme to apohemoglobin immobilized on the gold surface of the prism (Figure 6). The passage of an intense laser beam through the prism and its interaction with the gold surface shifted the SPR angle when heme was associated with the apohemoglobin compared to the angle observed for apohemoglobin alone. The biosensor was highly sensitive, required no heme labeling, and allowed real-time measurement. Furthermore, since the shift in the SPR angle was proportional to the heme concentration, the SPR biosensor enabled quantification of heme. The limit of detection was lower than 2 μM. This limit of detection is biologically relevant since biological heme levels in hemolytic pathological conditions vary between 1 and 50 μM.

**Figure 7:**
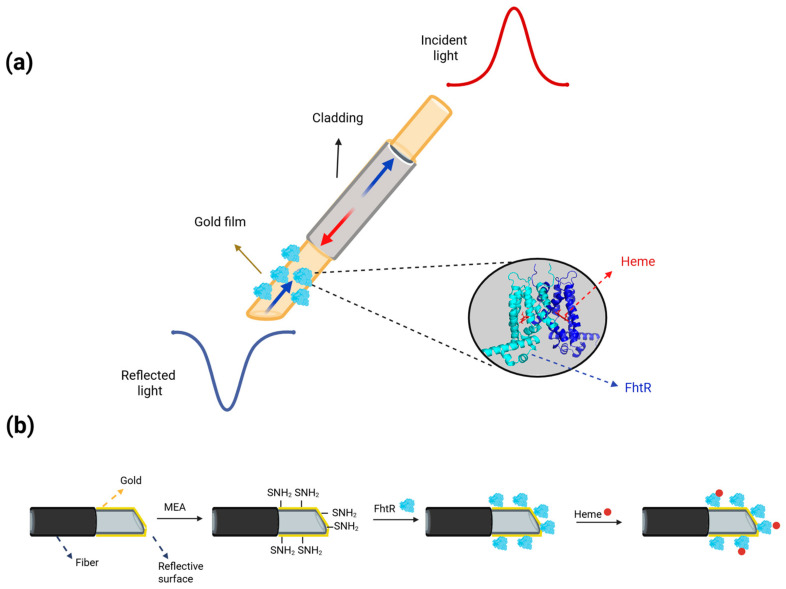
Principle of the fiber-optical SPR biosensor for heme detection. (**a**) The schematic diagram of an optic fiber-typed sensor; (**b**) surface modification of the optical fiber sensor. A plastic-clad optical fiber was coated with gold film deposition to excite the SPR effect. The sensor was immersed in a MEA solution to form an Au-S covalent bond between the gold layer and the -HS group in MEA. FhtR protein was then attached to a layer of free amino functional groups formed on the surface of the gold film of the sensor through the formation of an acylamino bond. Illustration adapted from [89] and created in BioRender. Vidic, J. (2025) https://BioRender.com/v1t2e74.

Recently, a label-free fiber-optical SPR heme biosensor was developed based on the specific biomolecular recognition between heme and its transport regulatory protein from *E. faecalis*, FhtR [89]. As mentioned in Section 4.4.2, FhtR binds intracellular heme molecules with high affinity [65] and was used as a sensing element, but its immobilization on the optical fiber required innovative surface chemistry. To attach FhtR to the optical fiber surface of the biosensor, the protein was modified with four aspartates at the C-terminus. The carboxyl functional groups of the aspartates covalently bound to the amino group of MEA molecules, which created a self-assembled monolayer on the gold surface, as illustrated in Figure 7.

Upon heme contact with the sensor surface, it binds to FhtR by coordinating with the central iron ion and accommodating the hydrophobic porphyrin ring within its hydrophobic binding pocket through van der Waals interactions. This binding event induces a measurable change in the local refractive index at the sensing interface, resulting in a detectable shift in the SPR wavelength. Consequently, heme concentration was quantitatively determined by monitoring the magnitude of the wavelength shift (Figure 7). Rapid determination of trace heme concentration was achieved, with a detection limit of 0.9 μg/mL and a response time of 18 min [89]. Moreover, the universality of the biosensor was confirmed in blood and marine water as detection environments.

### 5.3. Electrochemical Biosensors for Heme Detection

An attractive approach to developing biosensors for heme detection was established following the original discovery by Sen’s group that hemin-binding DNA aptamers can enhance the peroxidase activity of hemin through G-quadruplex structures [90]. This finding, that DNA aptamers for heme (Fe(III)-protoporphyrin IX complex) exhibit peroxidase and peroxygenase activities, has spurred extensive research into biosensors utilizing the G-quadruplex–hemin complex as a DNAzyme [91,92]. The G-quadruplex is a non-canonical DNA oligomer composed of four-stranded tertiary motifs formed in guanine-rich sequences [93,94]. This three-dimensional structure interacts strongly with hemin, which has been repeatedly shown to boost peroxidase activity beyond the original aptamer–hemin system. In particular, the binding of heme to the G-quadruplex requires a G-quartet motif formed by four guanines, but not an A-quartet formed by four adenines [95].

**Figure 8:**
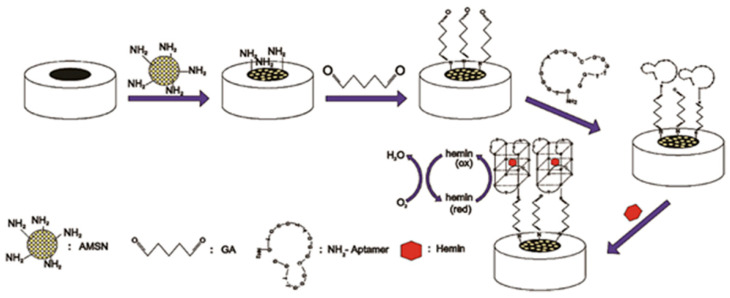
Electrochemical aptasensor for heme detection. Schematic diagram of steps of the electrode modification with amino-aptamer and hemin detection. Adapted with permission from [96].

Shekari et al. [96] constructed a label-free electrochemical aptasensor for the detection of hemin and hemoglobin (Hb). The sensor was fabricated by covalently immobilizing a hemin-specific G-quadruplex aptamer modified with an amino group onto a modified glassy carbon electrode using glutaraldehyde as a cross-linking agent. Upon exposure to hemin, a distinct peak current was observed, resulting from the formation of the aptamer–hemin complex (Figure 8). The peak intensity was proportional to the concentrations of heme and Hb. The limits of detection obtained using differential pulse voltammetry were as low as 7.5 × 10^−20^ M and 6.5 × 10^−20^ M for hemin and Hb, respectively.

Despite many advantages, the use of the aptamer–hemin complex for constructing aptasensors remains challenging. For instance, hemin can bind non-specifically to G-quadruplex aptamers, but the complex may lose target recognition [97]. In addition, the correct three-dimensional configuration of the aptamer, which significantly influences its binding performance with the target, can be compromised in complex biological matrices [93,98]. These drawbacks hinder the development of simple and broadly applicable aptasensors for heme detection [90]. Recently, Li et al. [99] extended the sequence of the terminal double-stranded domain of the hemin aptamer to stabilize its spatial conformation. By replacing the original G-quadruplex aptamer with this new optimized non-G-quadruplex aptamer, the resulting aptamer/hemin complex achieved 200% DNAzyme activity. High-sensitivity visual heme detection was demonstrated in both diluted serum and environmental water samples. Moreover, the optimized aptamer also distinguished multiple hemin analogs.

Interestingly, bacterial biofilms contain these non-canonical G-quadruplex DNA structures, whose functional roles are still poorly understood. A recent electrochemical study revealed that G-quadruplex DNA complexed with hemin enables extracellular electron transfer in biofilms [100]. This study reveals a previously unknown mechanism used by bacterial cells to conserve energy under oxygen limitation, and potentially also in their defense against oxidative stress during infection.

Electrochemical methods hold significant promise for heme detection in clinical settings, particularly for developing next-generation quantitative, portable, and low-cost point-of-care devices. Research is intense, focusing on overcoming the key drawbacks of selectivity, fouling, and reproducibility through advanced materials and sensor design [94]. However, widespread clinical adoption has not yet been realized. The major hurdles are less about the fundamental electrochemical detection and more about engineering a system that works reliably with minimal sample processing in complex real-world samples and proving its clinical and economic value over existing methods and the transition from a successful lab prototype to an FDA/CE-approved, commercially viable clinical device remains challenging.

### 5.4. Cell-Based Biosensors for Heme Detection

Measuring labile heme under hemolytic conditions is particularly challenging. Fluorescence-based optical biosensors, although requiring sample labeling with fluorescent tags, enable sensitive quantification of free heme within a single cell. Such biosensors measure changes in fluorescence intensity upon heme binding to the labeled recognition molecule. In many pathology studies, recognition is achieved through heme interaction with specific proteins. These molecules, such as antibodies directed against hemoglobin or substrates for peroxidase activity [101], can serve as specific recognition elements. For instance, Abshire et al. [102] used the genetically encoded fluorescence-based heme biosensor CHY to quantify cytosolic labile heme levels in intact, blood-stage *Plasmodium falciparum*. In this assay, fluorescent CHY encapsulated in giant multilamellar vesicles allowed quantification of cytosolic labile heme in *P. falciparum*. The binding of heme to CHY caused fluorescence quenching, which was measured using fluorescence microscopy imaging and fluorimetry. They found that during extensive degradation of host hemoglobin by the parasite, its own labile heme pool (~1.6 µM) was stably maintained. Because the biosensor was used in live parasites, it enabled determination of both labile heme levels and heme subcellular distribution. However, most heme sensors employing heme-dependent enzymatic fluorescent or colorimetric reporters, such as L-tryptophan-2,3-dioxygenase, ALAS, or HRP, require cell lysis and thus cannot be applied to single cells or used to determine heme subcellular localization [103,104]. In another study, Xu et al. [105] used the antimalarial drug artemisinin to develop a labile heme sensor and successfully applied it in cells and mice to quantify heme pools. Upon binding heme, the endoperoxide bridge in artemisinin was activated by its ferrous iron (Fe^2+^), and this reaction was used to achieve a fluorescent probe highly selective for labile heme without interference from hemin, protein-bound heme, or zinc protoporphyrin.

**Figure 9:**
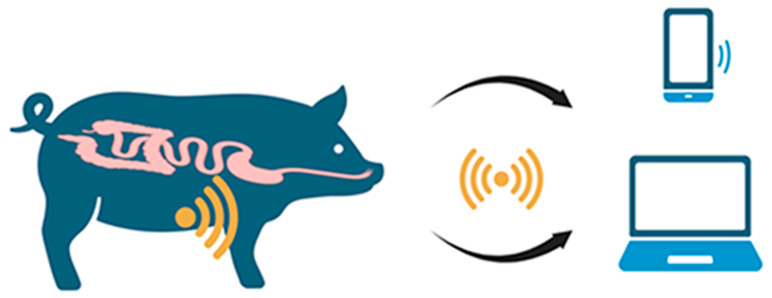
Ingestible micro-bio-electronic device (IMBED) for heme in vivo monitoring. The swine intestine was colonized with a transformed probiotic *E. coli* strain that senses heme due to the heterologous expression of the *Lactococcus lactis* heme-responsive transcriptional repressor (HrtR protein). After the microelectronics-equipped capsule deposition the heme level was transmitted to a laptop or a cellular phone. Illustration adapted from [106] and created in BioRender. Vidic, J. (2025) https://BioRender.com/2zu2dh5.

Mimee et al. [106] developed whole-cell biosensor bacteria and combined them with a miniaturized wireless readout capsule for in vivo biosensing of gastrointestinal bleeding. They applied synthetic biology to engineer a probiotic *E. coli* strain with complex genetic circuits to sense heme. The circuit consisted of a synthetic promoter (PL(HrtO)), regulated by the *Lactococcus lactis* heme-responsive transcriptional repressor (HrtR protein), and the outer-membrane transporter (ChuA), which enables the transit of extracellular heme through the bacterial envelope. Owing to their robust functionality and ability to survive and multiply in the gut, this cell-based biosensor measured gastrointestinal bleeding in real time. The signals were generated by the ingestible luminometer capsule, which was equipped with ultra-low-power microelectronics for wireless signal transmission (Figure 9).

**Figure 10:**
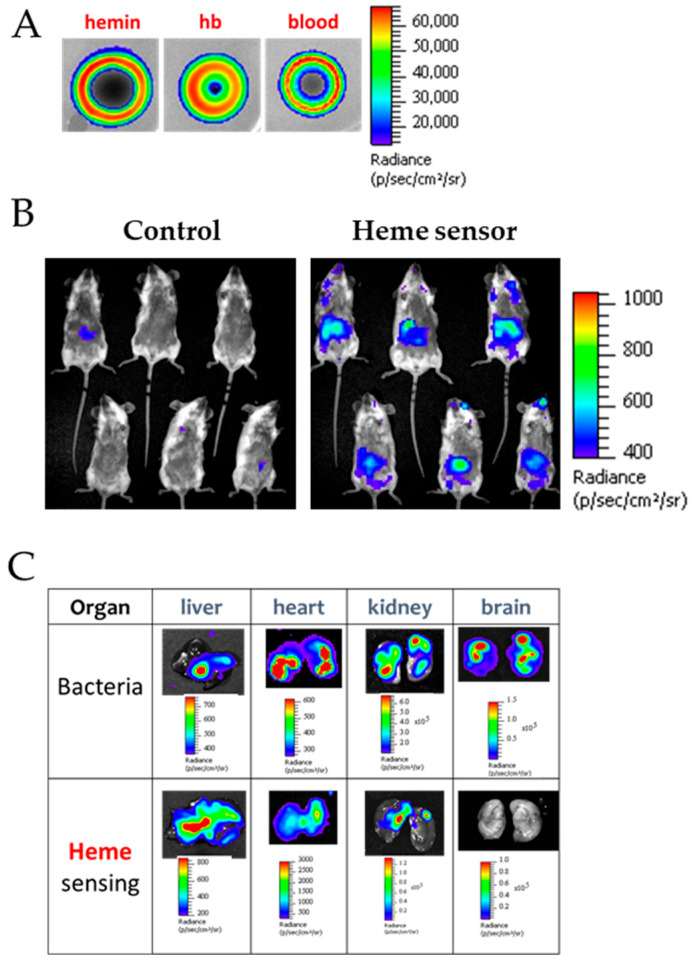
Bioluminescent heme sensing by *Streptococcus agalactiae* during the course of a systemic infection in mice. (**A**) Heme-dependent light emission by the heme-sensing *Streptococcus agalactiae* strain. Bacteria were transformed with a plasmid carrying the promoter of a heme-inducible promoter controlling the *luxABCDE* operon (heme-sensing plasmid). Culture of this strain was resuspended in soft agar and overlaid on agar plates. Hemin, or fresh mouse blood, was directly spotted on plates and luminescence visualized after 24 h using an IVIS 200 luminescence imaging system. (**B**) Heme sensing by *Streptococcus agalactiae* over the course of infection. Mice were infected with the heme biosensing bacteria transformed with the heme-responsive bioluminescent plasmid or a negative control plasmid. At 20 h post-infection, anesthetized mice were imaged in the IVIS 200 system. (**C**) Heme sensing versus bacterial load in dissected organs. Photographs and overlap images of photographs and luminescence are shown of different organs of mice infected either with a constitutively bioluminescent strain indicative of bacterial load (bacteria) or a heme-responsive strain (heme-sensing). Heme detection intensity by the heme sensing system is distinct in the different organs, indicative of differences in heme availability. Adapted with permission from [108].

Passage in the blood is a crucial step for colonization and pathogenesis of numerous pathogens. Luminescent heme whole-cell biosensor bacteria were engineered to monitor the capacity of pathogens to scavenge host heme during systemic infection while controlling its toxicity. A heme-responsive promoter controlling the *luxABCDE* operon from *Photorhabdus luminescens* [107] was inserted in *Streptococcus agalactiae*. The bacteria emitted light specifically in the presence of labile heme, heme hemoglobin, and blood (Figure 10A) [108]. The main advantage of the *luxABCDE* system is that it does not require the addition of an exogenous substrate, allowing direct observation of emitted luminescence in animal infection models in vivo using the IVIS imaging system (Figure 10B) [108]. Based on the heme sensor protein, pathogens such as *S. agalactiae* were shown to respond to host heme in vivo preferentially in specific organs, which contrasts with the overall pathogen distribution in the host (Figure 10C).

## 6. Conclusions and Perspectives

This review has examined the structure and function of heme, as well as the wide range of analytical methodologies developed to detect and quantify it. Although heme is harmless when tightly bound within hemoproteins, its release into the labile, unbound form can cause significant cellular and tissue damage, driving pro-inflammatory, oxidative, and hemolytic processes. Distinguishing protein-bound from free heme is therefore essential for understanding both physiological homeostasis and pathological conditions. Various instrumental approaches, including HPLC, mass spectrometry, and UV-visible spectroscopy, offer high sensitivity and specificity for labile heme detection. However, these techniques are time-consuming, require extensive sample preparation, and depend on sophisticated instrumentation, limiting their use for real-time, in vivo, or point-of-care applications.

In bacterial systems, heme acts as both a critical cofactor and a potent toxin, necessitating tight regulatory mechanisms. Gram-positive bacteria, in particular, rely on dedicated heme biosensor proteins to monitor and buffer intracellular heme levels, ensuring survival during hemolytic infections. These biosensors have also become powerful tools for probing heme homeostasis in living organisms. Advances in synthetic biology are now enabling the engineering of bacterial strains that combine heme-responsive proteins with fluorescent or chemiluminescent reporters, paving the way for innovative whole-cell biosensors relevant to both basic research and disease monitoring.

The trajectory of heme biosensing is shifting from simple quantification toward integrated, sample-to-answer microsystems. Key future directions include the development of in vivo or swallowable sensors for continuous gastrointestinal monitoring, and the creation of robust point-of-care devices that leverage smartphone readouts and automated microfluidic sample preparation, particularly for overcoming the profound matrix challenges of stool and blood. However, clinical translation remains bottlenecked by the sample preparation barrier, requiring on-device homogenization and purification, and by the need for large-scale clinical validation to prove utility against entrenched, low-cost standards like the fecal immunochemical test. Heme quantification is often compromised by (i) tight binding to endogenous proteins (albumin, hemopexin, hemoglobin fragments), which shields labile heme from detection; (ii) redox cycling and chemical instability, which modify heme speciation and affect redox-sensitive probes; (iii) non-specific interactions with lipids and membranes that partition or sequester heme; and (iv) matrix components that quench or amplify optical signals, including bilirubin, porphyrins, and particulate matter. Overcoming these interferences is a prerequisite for translating heme sensors from controlled buffer conditions to real biological samples.

Looking ahead, the next generation of heme detection systems will likely emerge at the interface of synthetic biology, advanced materials, and miniaturized analytical technologies. Integrating selective recognition with microfluidics, developing robust sensors resistant to matrix interference, and engineering whole-cell or cell-free biosensors capable of real-time reporting represent promising avenues. Such advances will be essential not only for basic research but also for clinical diagnostics, infection monitoring, and a broader understanding of heme-driven pathophysiology.

**Table 1:** Examples of methods for the detection of free heme.

Methods	Molecule Quantified	Sensitivity	Matrix	Comments	References
FOBT ^a^	Heme	Not determined	Feces	Colorimetric test based on heme peroxidase activity	[109]
iFOBT ^b^	Hemoglobin	≥10 μg/g	Feces	Quantitative test using hemoglobin antibodies fixed on latex beads	[110]
Urine test strips	Heme	0.15–0.45 mg/L	Urine	Colorimetric test based on heme peroxidase activity	[111]
Fluorescent probe	Labile heme	Not determined	Cells and mouse	Artemisinin fluorophore	[105]
FRET ^c^	Labile heme	10 nM	Buffer	Fluorescence of Cytochrome b-GFP decreases upon binding heme	[112]
FRET ^c^	Labile heme	Not determined	Buffer	Fluorescently labeled HO-1 was used as a recognition element	[87]
FRET ^c^	Labile heme	Not determined	Buffer	Peptide derived from hemoproteins was used as a fluorescent probe	[113]
RP–HPLC ^d^	Hemoprotein	Not determined	Urine, feces and blood	Detection of the porphyrin’s fluorescence at 600–630 nm	[114]
DWF–HPLC ^e^	Porphyrins	25 µM	Bood		[115]
ELISA	Hemoproteins	>0.15 µM	Plasma (mouse) HeLa Cells	Single domain antibodies (sdAbs) coupled with ELISA	[101]
Pyridine hemochromogen test	Hemoproteins	1 µM	Alkaline medium	Heme titration with pyridine used as a ligand	[116]
QuantiChrom assay Kit	Hemoproteins	0.5 nM	Buffer	Colorimetric test	[74]
Cysteine probe	Labile heme	0.5 µM	Plasma/human serum	Method distinguished between hemoglobin and free heme	[76]
Spectral deconvolution	Labile and bound heme	2 µM	Plasma	Spectrum decomposition to distinguish between oxyhemoglobin, methemoglobin and free heme	[117,118]
TA Microscopy ^f^	Hemoproteins	9.2 µM	*C. elegans* and mammalian cell lines	Visualization of heme uptake and subcellular localization of heme	[119]
rRaman ^g^	Hemoproteins	5 µM	HEK293 Cells	Patch-clamp system	[82]
SERS ^h^	Labile heme and its degradation products	20–100 µM	Buffer	Molecule excitation at several wavelengths to compare obtained data to a standard curve	[83]
SPR ^i^	Labile heme	2 µM	Buffer	Ligand-receptor measurement: apo-hemoglobin immobilization to a matrix	[88]
MALDI TOF–MS ^j^	Labile heme	400 nM	Blood Agar medium	MS techniques are very specific but are more efficient for heme detection than heme quantification	[120]
ESI–MS ^k^	Hemozoin (heme aggregate)/labile heme	100 nM	*P. falciparum* extract		[121]
HPLC-MS/MS ^l^	Labile heme/porphyrins	0.2 pM	Biological media and *E. coli*	HPLC optimization	[77]
RP–HPLC ^d^/UV-visible	Labile heme/porphyrins	Not determined	Biological medium and tissues	Chromatograms comparison between the sample and the standard	[122]
UPLC ^m^	Heme/hemozoin	Not determined	Spinal fluid, mouse tissue and *P. falciparum* extract	Simplest HPLC optimization	[123] [124]
IMBED ^n^	Labile heme	32.5 ppm	Biological sample	Bacterial biosensor coupled to an electronic system	[106]
HRP peroxidase activity method	Hemoproteins	5.6 nM–0.2 µM	Urine	Method using capillary electrophoresis coupled with chemiluminescence	[125]
HRP activity method	Labile heme	20 pM	Cellular medium	Measurement of the HRP allo-enzyme luminescence	[126]
		0.65 pM		Optimization	[75]
**HemoQuant** **(HRP activity method)**	Labile heme	>1 nM	Biological sample	Heme porphyrin excitation (λ_ex_ = 400 nm et λ_em_ = 662 nm)	[127]
**Electrochemical**	Heme and Hb	7.5 × 10^−20^ M heme 6.5 × 10^−20^ M Hb	Blood samples	G-quadruplex aptamer used as a sensing element	[96]
Electrochemical	Heme	0.9 μg/mL	Blood sample	FhtR as a sensing element	[89]
Electrochemical	Hemin	0.64 nM	Serum	G-quadruplex aptamer employed	[128]
Colorimetric	Hemin	0.1 nM	Serum and environmental water	Non-G-quadruplex aptamer was optimized	[99]

^a^ Fecal occult blood test; ^b^ Immunological fecal occult blood test; ^c^ Fluorescence Resonance Energy Transfer; ^d^ Reverse-Phase High-Performance Liquid Chromatography; ^e^ Dual wavelength fluorescence High-Performance Liquid Chromatography; ^f^ Transient Absorption Microscopy; ^g^ Resonance Raman Spectroscopy; ^h^ Surface Enhanced Raman Spectroscopy; ^i^ Surface Plasma Resonance; ^j^ Matrix-Assisted Laser Desorption/Ionization Time-of-Flight Mass Spectrometry; ^k^ Electrospray Ionization Mass Spectrometry; ^l^ High-Performance Liquid Chromatography—Tandem Mass Spectrometry, ^m^ Ultra-Performance Liquid Chromatography; ^n^ Ingestible Micro-Bio-Electronic Device.

## Data Availability

Not applicable.

## Abbreviations

The following abbreviations are used in this manuscript:

AA	Amino Acid
ALA	AminoLevulinic Acid
ALAS	AminoLevulinic Acid Synthase
CHY	α-Chymotrypsin
CO	Carbon monoxide
CO_2_	Carbon dioxide
DAMPs	Damage-Associated Molecular Patterns
DHp	Dimerization histidine phosphotransfer
DNA	DeoxyRibonucleic Acid
DWF	Dual Wavelength Fluorescence
FhtR	Faecalis heme transport Regulator
FOBT	Fecal Occult Blood Test
FRET	Fluorescence Resonance Energy Transfer
HAMP	Histidine kinase, Adenylate cyclase, Methyl-accepting protein and Phosphatase domain
HatR	Heme-activated transporter Regulator
*hatRT*	Heme-activated transporter Regulator/ Transporter operon
Hb	Hemoglobin
Hp	Haptoglobin
HPLC	High Performance Liquid Chromatography
HO-1	Heme-Oxygenase 1
HRP	HorseRadish Peroxidase
HrtBA	Heme-regulated transporter
HrtR	Heme-regulated transcriptional Regulator
HsmR	Heme-sensing membrane Regulator
*hsmRA*	Heme-sensing membrane protein system
HssS	Heme sensor system Sensor
HssR	Heme sensor system Regulator
Hx	Hemopexin
iFOBT	Immunological Fecal Occult Blood Test
IL	Interleukin
IMBED	Ingestible Micro-Bio-Electronic Device
MALDI-TOF	Matrix-Assisted Laser Desorption/Ionization Time-of-Flight
MarR	Multiple antibiotic resistance Regulator
MFS	Major Facilitator Superfamily
MS	Mass Spectrometry
NFkB	Nuclear Factor-kappa B
NO	Nitric Oxide
O_2_	Dioxygen
PAA	PolyAcrylic Acid
PCT	Porphyria Cutanea Tarda
Pef	Porphyrin efflux
PefAB	Porphyrin efflux transporter AB
PefCD	Porphyrin efflux transporter CD
PefR	Porphyrin efflux Regulator
PBG	Porphobilinogen
PPIX	Protoporphyrin IX
ROS	Reactive Oxygen Species
SCD	Sickle Cell Disease
SPR	Surface Plasmon Resonance
SERS	Surface-Enhanced Raman Scattering
TA Microscopy	Transient Absorption Microscopy
TetR	Tetracycline Repressor
TNF	Tumor Necrosis Factor
TLR4	Toll-Like Receptor 4
RP-HPLC	Reverse Phase-High Performance Liquid Chromatography
UPLC	Ultra Performance Liquid Chromatography
